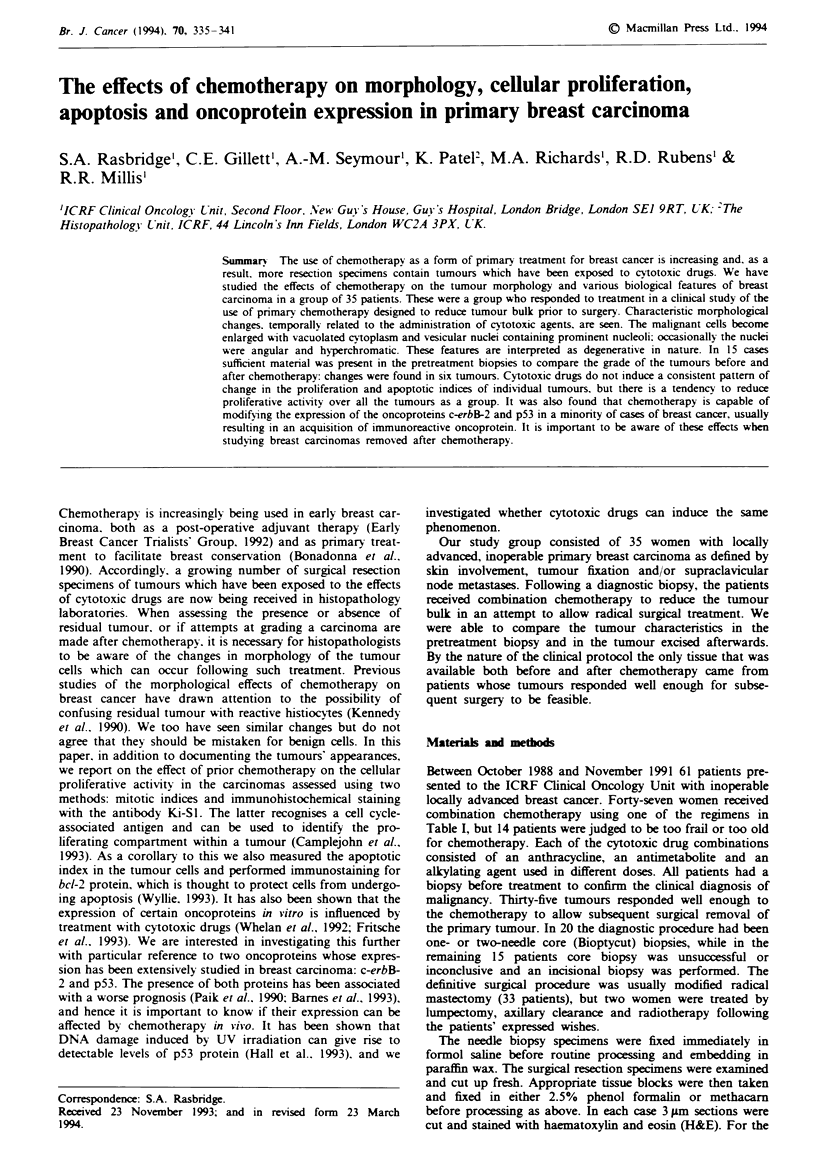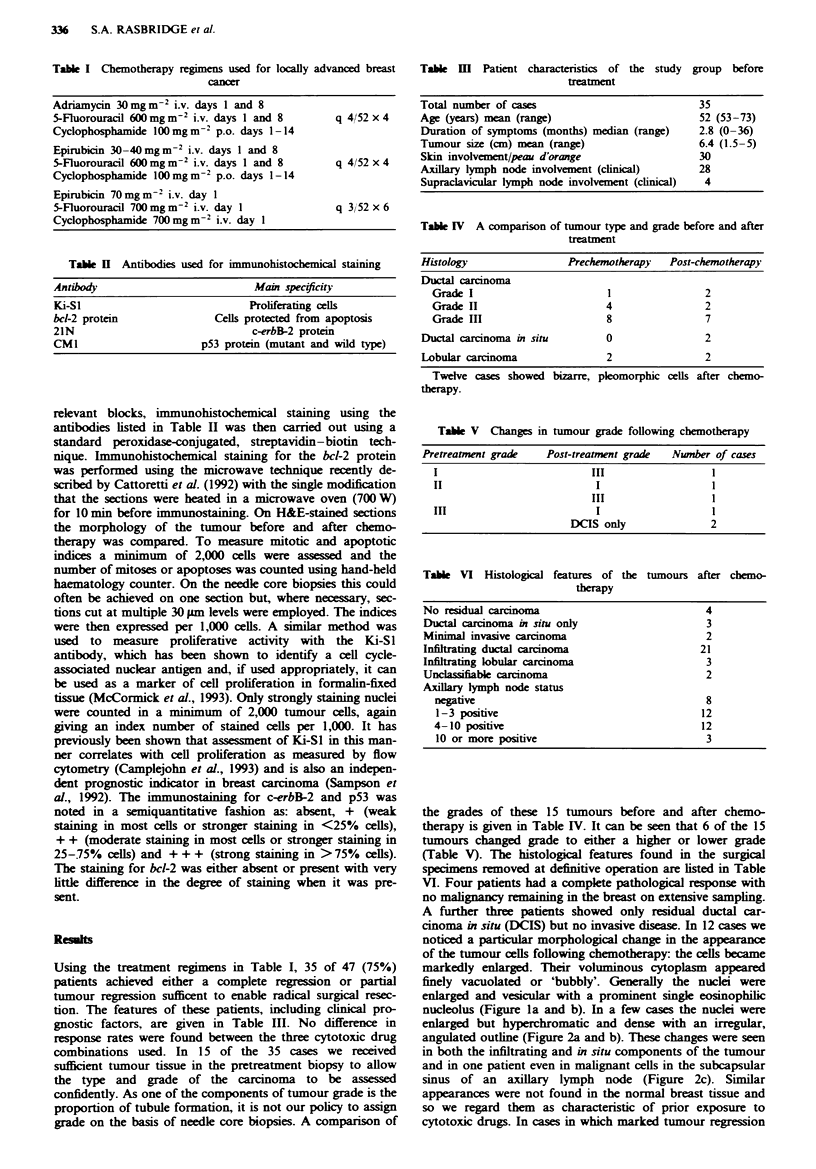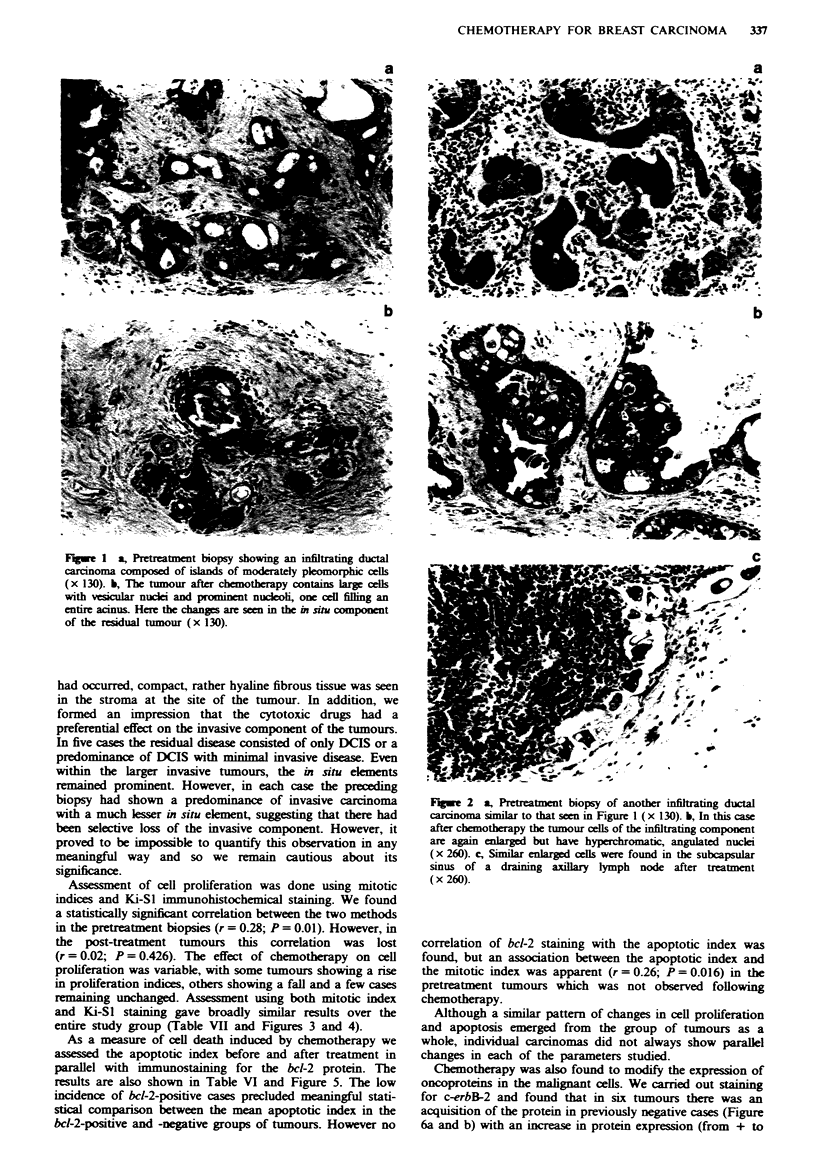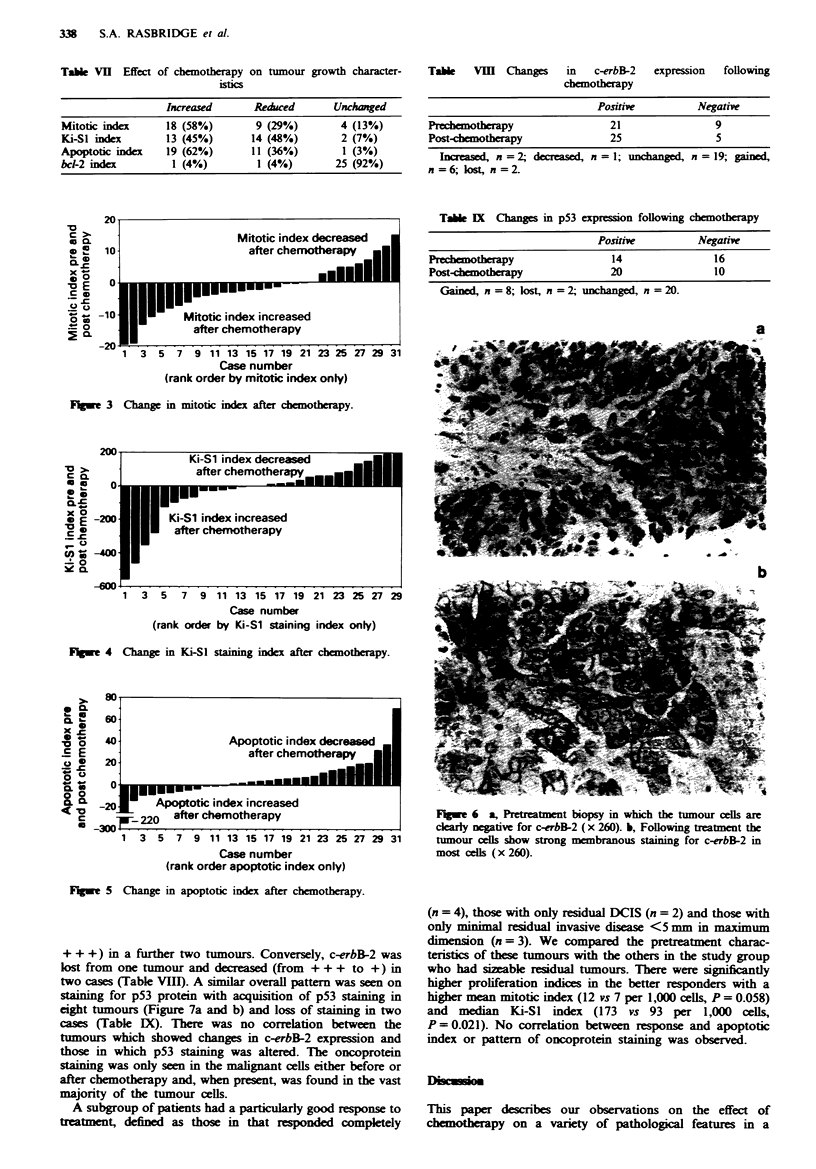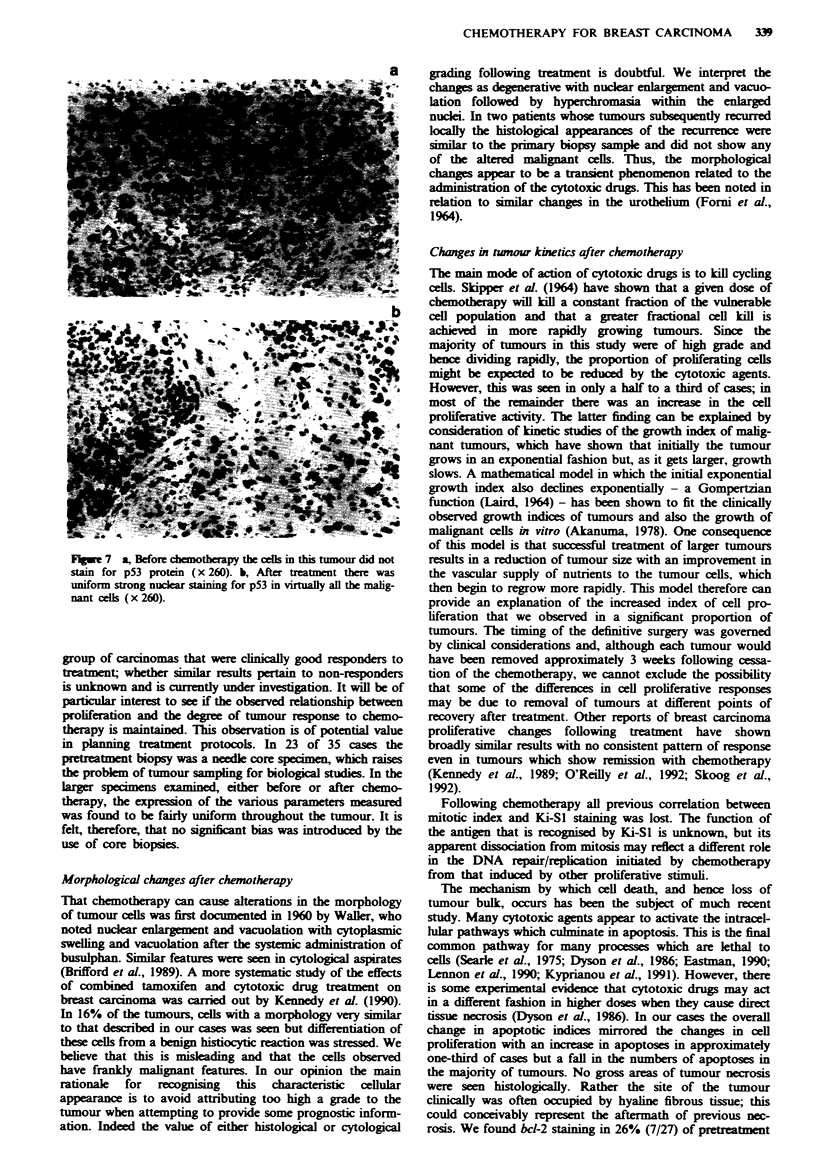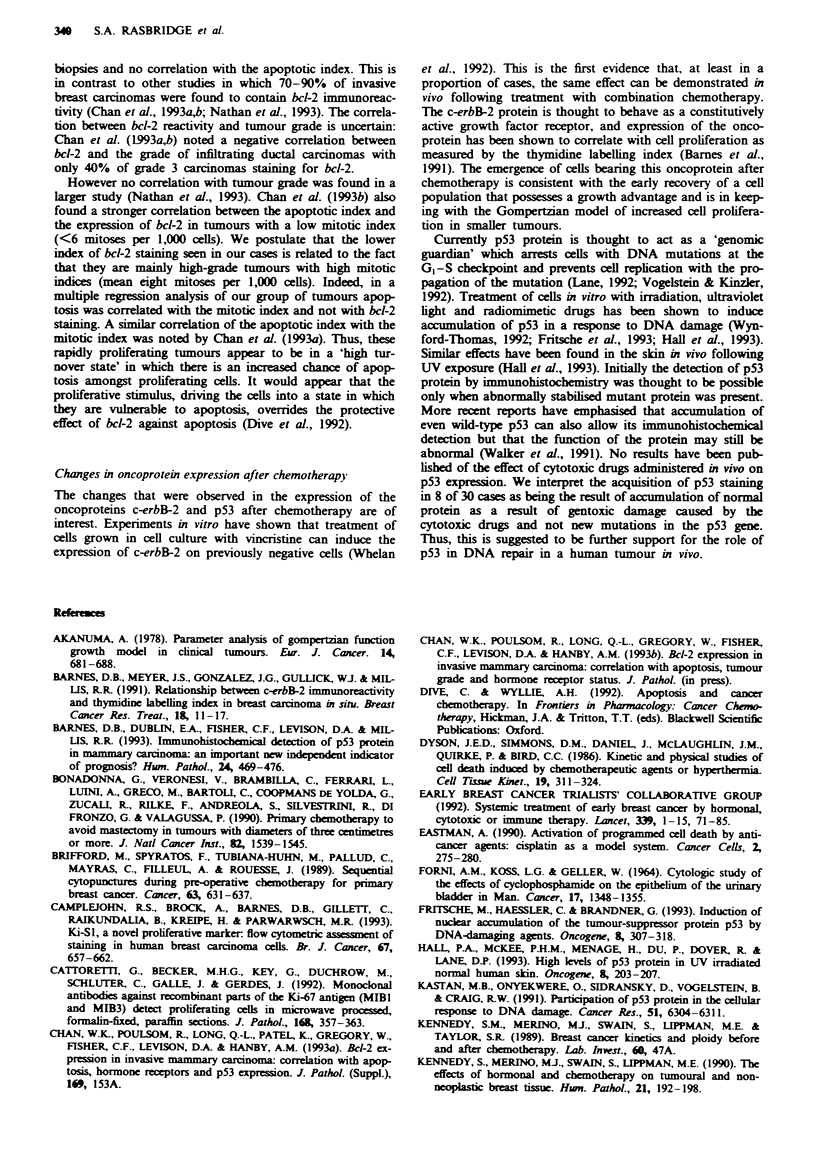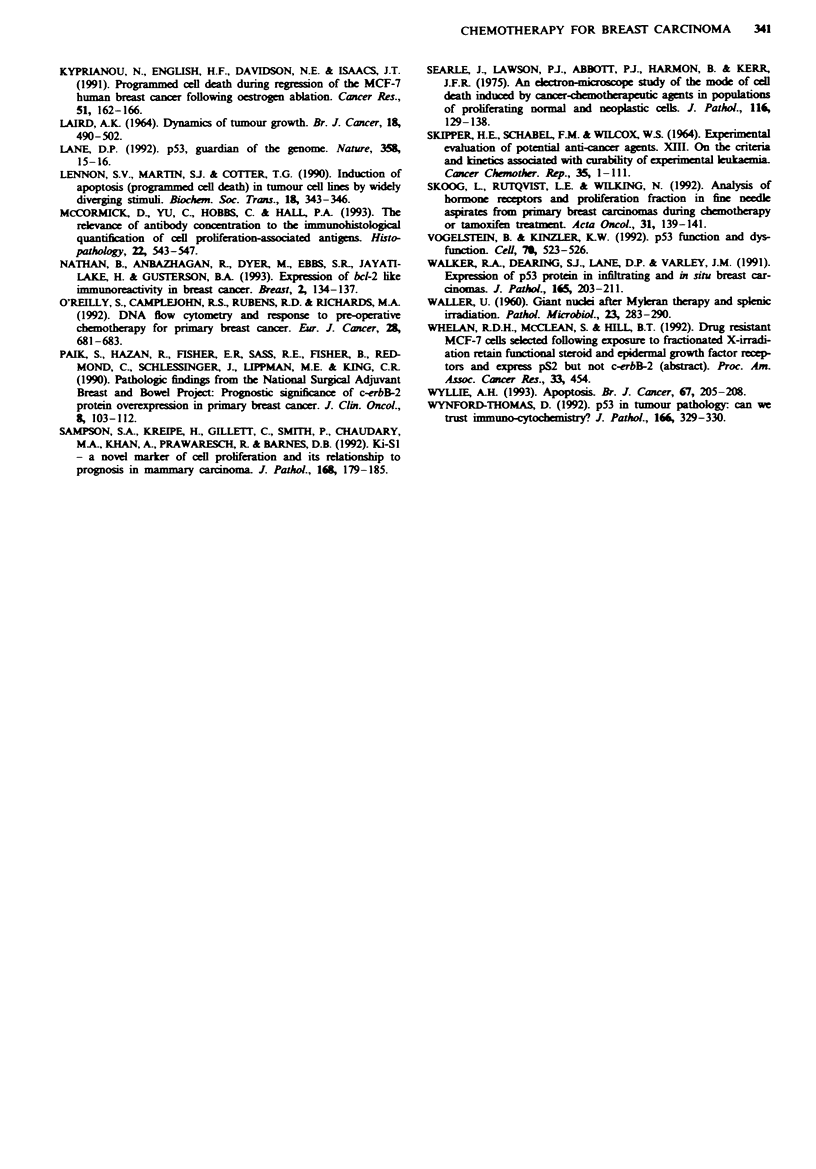# The effects of chemotherapy on morphology, cellular proliferation, apoptosis and oncoprotein expression in primary breast carcinoma.

**DOI:** 10.1038/bjc.1994.303

**Published:** 1994-08

**Authors:** S. A. Rasbridge, C. E. Gillett, A. M. Seymour, K. Patel, M. A. Richards, R. D. Rubens, R. R. Millis

**Affiliations:** ICRF Clinical Oncology Unit, Guy's Hospital, London, UK.

## Abstract

**Images:**


Br. J. Cancer (1994), 70, 335-341 © Macmillan Press Ltd., 1994

### The effects of chemotherapy on morphology, cellular proliferation, apoptosis and oncoprotein expression in primary breast carcinoma

S.A. Rasbridge[1], C.E. Gillett[1], A.-M. Seymour[1], K. Patel[2], M.A. Richards[1], R.D. Rubens[1] & R.R. Millis[1]

[1]*ICRF Clinical Oncology Unit, Second Floor, New Guy's House, Guy's Hospital, London Bridge, London SE1 9RT, UK;* [2]*The Histopathology Unit, ICRF, 44 Lincoln's Inn Fields, London WC2A 3PX, UK.*

**Summary** The use of chemotherapy as a form of primary treatment for breast cancer is increasing and, as a result, more resection specimens contain tumours which have been exposed to cytotoxic drugs. We have studied the effects of chemotherapy on the tumour morphology and various biological features of breast carcinoma in a group of 35 patients. These were a group who responded to treatment in a clinical study of the use of primary chemotherapy designed to reduce tumour bulk prior to surgery. Characteristic morphological changes, temporally related to the administration of cytotoxic agents, are seen. The malignant cells become enlarged with vacuolated cytoplasm and vesicular nuclei containing prominent nucleoli; occasionally the nuclei were angular and hyperchromatic. These features are interpreted as degenerative in nature. In 15 cases sufficient material was present in the pretreatment biopsies to compare the grade of the tumours before and after chemotherapy: changes were found in six tumours. Cytotoxic drugs do not induce a consistent pattern of change in the proliferation and apoptotic indices of individual tumours, but there is a tendency to reduce proliferative activity over all the tumours as a group. It was also found that chemotherapy is capable of modifying the expression of the oncoproteins c-*erb*B-2 and p53 in a minority of cases of breast cancer, usually resulting in an acquisition of immunoreactive oncoprotein. It is important to be aware of these effects when studying breast carcinomas removed after chemotherapy.

Chemotherapy is increasingly being used in early breast carcinoma, both as a post-operative adjuvant therapy (Early Breast Cancer Trialists' Group, 1992) and as primary treatment to facilitate breast conservation (Bonadonna *et al.*, 1990). Accordingly, a growing number of surgical resection specimens of tumours which have been exposed to the effects of cytotoxic drugs are now being received in histopathology laboratories. When assessing the presence or absence of residual tumour, or if attempts at grading a carcinoma are made after chemotherapy, it is necessary for histopathologists to be aware of the changes in morphology of the tumour cells which can occur following such treatment. Previous studies of the morphological effects of chemotherapy on breast cancer have drawn attention to the possibility of confusing residual tumour with reactive histiocytes (Kennedy *et al.*, 1990). We too have seen similar changes but do not agree that they should be mistaken for benign cells. In this paper, in addition to documenting the tumours' appearances, we report on the effect of prior chemotherapy on the cellular proliferative activity in the carcinomas assessed using two methods: mitotic indices and immunohistochemical staining with the antibody Ki-S1. The latter recognises a cell cycle-associated antigen and can be used to identify the proliferating compartment within a tumour (Camplejohn *et al.*, 1993). As a corollary to this we also measured the apoptotic index in the tumour cells and performed immunostaining for *bcl*-2 protein, which is thought to protect cells from undergoing apoptosis (Wyllie, 1993). It has also been shown that the expression of certain oncoproteins *in vitro* is influenced by treatment with cytotoxic drugs (Whelan *et al.*, 1992; Fritsche *et al.*, 1993). We are interested in investigating this further with particular reference to two oncoproteins whose expression has been extensively studied in breast carcinoma: c-*erb*B-2 and p53. The presence of both proteins has been associated with a worse prognosis (Paik *et al.*, 1990; Barnes *et al.*, 1993), and hence it is important to know if their expression can be affected by chemotherapy *in vivo*. It has been shown that DNA damage induced by UV irradiation can give rise to detectable levels of p53 protein (Hall et al., 1993), and we investigated whether cytotoxic drugs can induce the same phenomenon.

Our study group consisted of 35 women with locally advanced, inoperable primary breast carcinoma as defined by skin involvement, tumour fixation and/or supraclavicular node metastases. Following a diagnostic biopsy, the patients received combination chemotherapy to reduce the tumour bulk in an attempt to allow radical surgical treatment. We were able to compare the tumour characteristics in the pretreatment biopsy and in the tumour excised afterwards. By the nature of the clinical protocol the only tissue that was available both before and after chemotherapy came from patients whose tumours responded well enough for subsequent surgery to be feasible.

### Materials and methods

Between October 1988 and November 1991 61 patients presented to the ICRF Clinical Oncology Unit with inoperable locally advanced breast cancer. Forty-seven women received combination chemotherapy using one of the regimens in Table I, but 14 patients were judged to be too frail or too old for chemotherapy. Each of the cytotoxic drug combinations consisted of an anthracycline, an antimetabolite and an alkylating agent used in different doses. All patients had a biopsy before treatment to confirm the clinical diagnosis of malignancy. Thirty-five tumours responded well enough to the chemotherapy to allow subsequent surgical removal of the primary tumour. In 20 the diagnostic procedure had been one- or two-needle core (Bioptycut) biopsies, while in the remaining 15 patients core biopsy was unsuccessful or inconclusive and an incisional biopsy was performed. The definitive surgical procedure was usually modified radical mastectomy (33 patients), but two women were treated by lumpectomy, axillary clearance and radiotherapy following the patients' expressed wishes.

The needle biopsy specimens were fixed immediately in formol saline before routine processing and embedding in paraffin wax. The surgical resection specimens were examined and cut up fresh. Appropriate tissue blocks were then taken and fixed in either 2.5% phenol formalin or methacarn before processing as above. In each case 3 μm sections were cut and stained with haematoxylin and eosin (H&E). For the

Correspondence: S.A. Rasbridge.
Received 23 November 1993; and in revised form 23 March 1994.

**Table I** Chemotherapy regimens used for locally advanced breast cancer

| | |
|---|---|
| Adriamycin 30 mg m$^{-2}$ i.v. days 1 and 8<br>5-Fluorouracil 600 mg m$^{-2}$ i.v. days 1 and 8<br>Cyclophosphamide 100 mg m$^{-2}$ p.o. days 1–14 | q 4/52 × 4 |
| Epirubicin 30–40 mg m$^{-2}$ i.v. days 1 and 8<br>5-Fluorouracil 600 mg m$^{-2}$ i.v. days 1 and 8<br>Cyclophosphamide 100 mg m$^{-2}$ p.o. days 1–14 | q 4/52 × 4 |
| Epirubicin 70 mg m$^{-2}$ i.v. day 1<br>5-Fluorouracil 700 mg m$^{-2}$ i.v. day 1<br>Cyclophosphamide 700 mg m$^{-2}$ i.v. day 1 | q 3/52 × 6 |

**Table II** Antibodies used for immunohistochemical staining

| *Antibody* | *Main specificity* |
|---|---|
| Ki-S1 | Proliferating cells |
| *bcl*-2 protein | Cells protected from apoptosis |
| 21N | c-*erb*B-2 protein |
| CM1 | p53 protein (mutant and wild type) |

relevant blocks, immunohistochemical staining using the antibodies listed in Table II was then carried out using a standard peroxidase-conjugated, streptavidin–biotin technique. Immunohistochemical staining for the *bcl*-2 protein was performed using the microwave technique recently described by Cattoretti *et al.* (1992) with the single modification that the sections were heated in a microwave oven (700 W) for 10 min before immunostaining. On H&E-stained sections the morphology of the tumour before and after chemotherapy was compared. To measure mitotic and apoptotic indices a minimum of 2,000 cells were assessed and the number of mitoses or apoptoses was counted using hand-held haematology counter. On the needle core biopsies this could often be achieved on one section but, where necessary, sections cut at multiple 30 µm levels were employed. The indices were then expressed per 1,000 cells. A similar method was used to measure proliferative activity with the Ki-S1 antibody, which has been shown to identify a cell cycle-associated nuclear antigen and, if used appropriately, it can be used as a marker of cell proliferation in formalin-fixed tissue (McCormick *et al.*, 1993). Only strongly staining nuclei were counted in a minimum of 2,000 tumour cells, again giving an index number of stained cells per 1,000. It has previously been shown that assessment of Ki-S1 in this manner correlates with cell proliferation as measured by flow cytometry (Camplejohn *et al.*, 1993) and is also an independent prognostic indicator in breast carcinoma (Sampson *et al.*, 1992). The immunostaining for c-*erb*B-2 and p53 was noted in a semiquantitative fashion as: absent, + (weak staining in most cells or stronger staining in <25% cells), + + (moderate staining in most cells or stronger staining in 25–75% cells) and + + + (strong staining in >75% cells). The staining for *bcl*-2 was either absent or present with very little difference in the degree of staining when it was present.

### Results

Using the treatment regimens in Table I, 35 of 47 (75%) patients achieved either a complete regression or partial tumour regression sufficent to enable radical surgical resection. The features of these patients, including clinical prognostic factors, are given in Table III. No difference in response rates were found between the three cytotoxic drug combinations used. In 15 of the 35 cases we received sufficient tumour tissue in the pretreatment biopsy to allow the type and grade of the carcinoma to be assessed confidently. As one of the components of tumour grade is the proportion of tubule formation, it is not our policy to assign grade on the basis of needle core biopsies. A comparison of

**Table III** Patient characteristics of the study group before treatment

| | |
|---|---|
| Total number of cases | 35 |
| Age (years) mean (range) | 52 (53–73) |
| Duration of symptoms (months) median (range) | 2.8 (0–36) |
| Tumour size (cm) mean (range) | 6.4 (1.5–5) |
| Skin involvement/*peau d'orange* | 30 |
| Axillary lymph node involvement (clinical) | 28 |
| Supraclavicular lymph node involvement (clinical) | 4 |

**Table IV** A comparison of tumour type and grade before and after treatment

| *Histology* | *Prechemotherapy* | *Post-chemotherapy* |
|---|---|---|
| Ductal carcinoma | | |
| Grade I | 1 | 2 |
| Grade II | 4 | 2 |
| Grade III | 8 | 7 |
| Ductal carcinoma *in situ* | 0 | 2 |
| Lobular carcinoma | 2 | 2 |

Twelve cases showed bizarre, pleomorphic cells after chemotherapy.

**Table V** Changes in tumour grade following chemotherapy

| *Pretreatment grade* | *Post-treatment grade* | *Number of cases* |
|---|---|---|
| I | III | 1 |
| II | I | 1 |
| | III | 1 |
| III | I | 1 |
| | DCIS only | 2 |

**Table VI** Histological features of the tumours after chemotherapy

| | |
|---|---|
| No residual carcinoma | 4 |
| Ductal carcinoma *in situ* only | 3 |
| Minimal invasive carcinoma | 2 |
| Infiltrating ductal carcinoma | 21 |
| Infiltrating lobular carcinoma | 3 |
| Unclassifiable carcinoma | 2 |
| Axillary lymph node status | |
| negative | 8 |
| 1–3 positive | 12 |
| 4–10 positive | 12 |
| 10 or more positive | 3 |

the grades of these 15 tumours before and after chemotherapy is given in Table IV. It can be seen that 6 of the 15 tumours changed grade to either a higher or lower grade (Table V). The histological features found in the surgical specimens removed at definitive operation are listed in Table VI. Four patients had a complete pathological response with no malignancy remaining in the breast on extensive sampling. A further three patients showed only residual ductal carcinoma *in situ* (DCIS) but no invasive disease. In 12 cases we noticed a particular morphological change in the appearance of the tumour cells following chemotherapy: the cells became markedly enlarged. Their voluminous cytoplasm appeared finely vacuolated or 'bubbly'. Generally the nuclei were enlarged and vesicular with a prominent single eosinophilic nucleolus (Figure 1a and b). In a few cases the nuclei were enlarged but hyperchromatic and dense with an irregular, angulated outline (Figure 2a and b). These changes were seen in both the infiltrating and *in situ* components of the tumour and in one patient even in malignant cells in the subcapsular sinus of an axillary lymph node (Figure 2c). Similar appearances were not found in the normal breast tissue and so we regard them as characteristic of prior exposure to cytotoxic drugs. In cases in which marked tumour regression

**Figure 1** **a**, Pretreatment biopsy showing an infiltrating ductal carcinoma composed of islands of moderately pleomorphic cells (× 130). **b**, The tumour after chemotherapy contains large cells with vesicular nuclei and prominent nucleoli, one cell filling an entire acinus. Here the changes are seen in the *in situ* component of the residual tumour (× 130).

**Figure 2** **a**, Pretreatment biopsy of another infiltrating ductal carcinoma similar to that seen in Figure 1 (× 130). **b**, In this case after chemotherapy the tumour cells of the infiltrating component are again enlarged but have hyperchromatic, angulated nuclei (× 260). **c**, Similar enlarged cells were found in the subcapsular sinus of a draining axillary lymph node after treatment (× 260).

had occurred, compact, rather hyaline fibrous tissue was seen in the stroma at the site of the tumour. In addition, we formed an impression that the cytotoxic drugs had a preferential effect on the invasive component of the tumours. In five cases the residual disease consisted of only DCIS or a predominance of DCIS with minimal invasive disease. Even within the larger invasive tumours, the *in situ* elements remained prominent. However, in each case the preceding biopsy had shown a predominance of invasive carcinoma with a much lesser *in situ* element, suggesting that there had been selective loss of the invasive component. However, it proved to be impossible to quantify this observation in any meaningful way and so we remain cautious about its significance.

Assessment of cell proliferation was done using mitotic indices and Ki-S1 immunohistochemical staining. We found a statistically significant correlation between the two methods in the pretreatment biopsies ($r = 0.28$; $P = 0.01$). However, in the post-treatment tumours this correlation was lost ($r = 0.02$; $P = 0.426$). The effect of chemotherapy on cell proliferation was variable, with some tumours showing a rise in proliferation indices, others showing a fall and a few cases remaining unchanged. Assessment using both mitotic index and Ki-S1 staining gave broadly similar results over the entire study group (Table VII and Figures 3 and 4).

As a measure of cell death induced by chemotherapy we assessed the apoptotic index before and after treatment in parallel with immunostaining for the *bcl*-2 protein. The results are also shown in Table VI and Figure 5. The low incidence of *bcl*-2-positive cases precluded meaningful statistical comparison between the mean apoptotic index in the *bcl*-2-positive and -negative groups of tumours. However no correlation of *bcl*-2 staining with the apoptotic index was found, but an association between the apoptotic index and the mitotic index was apparent ($r = 0.26$; $P = 0.016$) in the pretreatment tumours which was not observed following chemotherapy.

Although a similar pattern of changes in cell proliferation and apoptosis emerged from the group of tumours as a whole, individual carcinomas did not always show parallel changes in each of the parameters studied.

Chemotherapy was also found to modify the expression of oncoproteins in the malignant cells. We carried out staining for c-*erb*B-2 and found that in six tumours there was an acquisition of the protein in previously negative cases (Figure 6a and b) with an increase in protein expression (from + to

**Table VII** Effect of chemotherapy on tumour growth characteristics

| | *Increased* | *Reduced* | *Unchanged* |
|---|---|---|---|
| Mitotic index | 18 (58%) | 9 (29%) | 4 (13%) |
| Ki-S1 index | 13 (45%) | 14 (48%) | 2 (7%) |
| Apoptotic index | 19 (62%) | 11 (36%) | 1 (3%) |
| *bcl*-2 index | 1 (4%) | 1 (4%) | 25 (92%) |

**Figure 3** Change in mitotic index after chemotherapy.

**Figure 4** Change in Ki-S1 staining index after chemotherapy.

**Figure 5** Change in apoptotic index after chemotherapy.

+ + +) in a further two tumours. Conversely, c-*erb*B-2 was lost from one tumour and decreased (from + + + to +) in two cases (Table VIII). A similar overall pattern was seen on staining for p53 protein with acquisition of p53 staining in eight tumours (Figure 7a and b) and loss of staining in two cases (Table IX). There was no correlation between the tumours which showed changes in c-*erb*B-2 expression and those in which p53 staining was altered. The oncoprotein staining was only seen in the malignant cells either before or after chemotherapy and, when present, was found in the vast majority of the tumour cells.

A subgroup of patients had a particularly good response to treatment, defined as those in that responded completely

**Table VIII** Changes in c-*erb*B-2 expression following chemotherapy

| | *Positive* | *Negative* |
|---|---|---|
| Prechemotherapy | 21 | 9 |
| Post-chemotherapy | 25 | 5 |

Increased, $n = 2$; decreased, $n = 1$; unchanged, $n = 19$; gained, $n = 6$; lost, $n = 2$.

**Table IX** Changes in p53 expression following chemotherapy

| | *Positive* | *Negative* |
|---|---|---|
| Prechemotherapy | 14 | 16 |
| Post-chemotherapy | 20 | 10 |

Gained, $n = 8$; lost, $n = 2$; unchanged, $n = 20$.

**Figure 6** **a**, Pretreatment biopsy in which the tumour cells are clearly negative for c-*erb*B-2 (×260). **b**, Following treatment the tumour cells show strong membranous staining for c-*erb*B-2 in most cells (×260).

($n = 4$), those with only residual DCIS ($n = 2$) and those with only minimal residual invasive disease <5 mm in maximum dimension ($n = 3$). We compared the pretreatment characteristics of these tumours with the others in the study group who had sizeable residual tumours. There were significantly higher proliferation indices in the better responders with a higher mean mitotic index (12 *vs* 7 per 1,000 cells, $P = 0.058$) and median Ki-S1 index (173 *vs* 93 per 1,000 cells, $P = 0.021$). No correlation between response and apoptotic index or pattern of oncoprotein staining was observed.

### Discussion

This paper describes our observations on the effect of chemotherapy on a variety of pathological features in a

**Figure 7** a, Before chemotherapy the cells in this tumour did not stain for p53 protein (× 260). b, After treatment there was uniform strong nuclear staining for p53 in virtually all the malignant cells (× 260).

group of carcinomas that were clinically good responders to treatment; whether similar results pertain to non-responders is unknown and is currently under investigation. It will be of particular interest to see if the observed relationship between proliferation and the degree of tumour response to chemotherapy is maintained. This observation is of potential value in planning treatment protocols. In 23 of 35 cases the pretreatment biopsy was a needle core specimen, which raises the problem of tumour sampling for biological studies. In the larger specimens examined, either before or after chemotherapy, the expression of the various parameters measured was found to be fairly uniform throughout the tumour. It is felt, therefore, that no significant bias was introduced by the use of core biopsies.

*Morphological changes after chemotherapy*

That chemotherapy can cause alterations in the morphology of tumour cells was first documented in 1960 by Waller, who noted nuclear enlargement and vacuolation with cytoplasmic swelling and vacuolation after the systemic administration of busulphan. Similar features were seen in cytological aspirates (Brifford *et al.*, 1989). A more systematic study of the effects of combined tamoxifen and cytotoxic drug treatment on breast carcinoma was carried out by Kennedy *et al.* (1990). In 16% of the tumours, cells with a morphology very similar to that described in our cases was seen but differentiation of these cells from a benign histiocytic reaction was stressed. We believe that this is misleading and that the cells observed have frankly malignant features. In our opinion the main rationale for recognising this characteristic cellular appearance is to avoid attributing too high a grade to the tumour when attempting to provide some prognostic information. Indeed the value of either histological or cytological grading following treatment is doubtful. We interpret the changes as degenerative with nuclear enlargement and vacuolation followed by hyperchromasia within the enlarged nuclei. In two patients whose tumours subsequently recurred locally the histological appearances of the recurrence were similar to the primary biopsy sample and did not show any of the altered malignant cells. Thus, the morphological changes appear to be a transient phenomenon related to the administration of the cytotoxic drugs. This has been noted in relation to similar changes in the urothelium (Forni *et al.*, 1964).

*Changes in tumour kinetics after chemotherapy*

The main mode of action of cytotoxic drugs is to kill cycling cells. Skipper *et al.* (1964) have shown that a given dose of chemotherapy will kill a constant fraction of the vulnerable cell population and that a greater fractional cell kill is achieved in more rapidly growing tumours. Since the majority of tumours in this study were of high grade and hence dividing rapidly, the proportion of proliferating cells might be expected to be reduced by the cytotoxic agents. However, this was seen in only a half to a third of cases; in most of the remainder there was an increase in the cell proliferative activity. The latter finding can be explained by consideration of kinetic studies of the growth index of malignant tumours, which have shown that initially the tumour grows in an exponential fashion but, as it gets larger, growth slows. A mathematical model in which the initial exponential growth index also declines exponentially – a Gompertzian function (Laird, 1964) – has been shown to fit the clinically observed growth indices of tumours and also the growth of malignant cells *in vitro* (Akanuma, 1978). One consequence of this model is that successful treatment of larger tumours results in a reduction of tumour size with an improvement in the vascular supply of nutrients to the tumour cells, which then begin to regrow more rapidly. This model therefore can provide an explanation of the increased index of cell proliferation that we observed in a significant proportion of tumours. The timing of the definitive surgery was governed by clinical considerations and, although each tumour would have been removed approximately 3 weeks following cessation of the chemotherapy, we cannot exclude the possibility that some of the differences in cell proliferative responses may be due to removal of tumours at different points of recovery after treatment. Other reports of breast carcinoma proliferative changes following treatment have shown broadly similar results with no consistent pattern of response even in tumours which show remission with chemotherapy (Kennedy *et al.*, 1989; O'Reilly *et al.*, 1992; Skoog *et al.*, 1992).

Following chemotherapy all previous correlation between mitotic index and Ki-S1 staining was lost. The function of the antigen that is recognised by Ki-S1 is unknown, but its apparent dissociation from mitosis may reflect a different role in the DNA repair/replication initiated by chemotherapy from that induced by other proliferative stimuli.

The mechanism by which cell death, and hence loss of tumour bulk, occurs has been the subject of much recent study. Many cytotoxic agents appear to activate the intracellular pathways which culminate in apoptosis. This is the final common pathway for many processes which are lethal to cells (Searle *et al.*, 1975; Dyson *et al.*, 1986; Eastman, 1990; Lennon *et al.*, 1990; Kyprianou *et al.*, 1991). However, there is some experimental evidence that cytotoxic drugs may act in a different fashion in higher doses when they cause direct tissue necrosis (Dyson *et al.*, 1986). In our cases the overall change in apoptotic indices mirrored the changes in cell proliferation with an increase in apoptoses in approximately one-third of cases but a fall in the numbers of apoptoses in the majority of tumours. No gross areas of tumour necrosis were seen histologically. Rather the site of the tumour clinically was often occupied by hyaline fibrous tissue; this could conceivably represent the aftermath of previous necrosis. We found *bcl*-2 staining in 26% (7/27) of pretreatment biopsies and no correlation with the apoptotic index. This is in contrast to other studies in which 70–90% of invasive breast carcinomas were found to contain *bcl*-2 immunoreactivity (Chan *et al*., 1993*a*,*b*; Nathan *et al*., 1993). The correlation between *bcl*-2 reactivity and tumour grade is uncertain: Chan *et al*. (1993*a*,*b*) noted a negative correlation between *bcl*-2 and the grade of infiltrating ductal carcinomas with only 40% of grade 3 carcinomas staining for *bcl*-2.

However no correlation with tumour grade was found in a larger study (Nathan *et al*., 1993). Chan *et al*. (1993*b*) also found a stronger correlation between the apoptotic index and the expression of *bcl*-2 in tumours with a low mitotic index (<6 mitoses per 1,000 cells). We postulate that the lower index of *bcl*-2 staining seen in our cases is related to the fact that they are mainly high-grade tumours with high mitotic indices (mean eight mitoses per 1,000 cells). Indeed, in a multiple regression analysis of our group of tumours apoptosis was correlated with the mitotic index and not with *bcl*-2 staining. A similar correlation of the apoptotic index with the mitotic index was noted by Chan *et al*. (1993*a*). Thus, these rapidly proliferating tumours appear to be in a 'high turnover state' in which there is an increased chance of apoptosis amongst proliferating cells. It would appear that the proliferative stimulus, driving the cells into a state in which they are vulnerable to apoptosis, overrides the protective effect of *bcl*-2 against apoptosis (Dive *et al*., 1992).

### *Changes in oncoprotein expression after chemotherapy*

The changes that were observed in the expression of the oncoproteins c-*erb*B-2 and p53 after chemotherapy are of interest. Experiments *in vitro* have shown that treatment of cells grown in cell culture with vincristine can induce the expression of c-*erb*B-2 on previously negative cells (Whelan *et al*., 1992). This is the first evidence that, at least in a proportion of cases, the same effect can be demonstrated *in vivo* following treatment with combination chemotherapy. The c-*erb*B-2 protein is thought to behave as a constitutively active growth factor receptor, and expression of the oncoprotein has been shown to correlate with cell proliferation as measured by the thymidine labelling index (Barnes *et al*., 1991). The emergence of cells bearing this oncoprotein after chemotherapy is consistent with the early recovery of a cell population that possesses a growth advantage and is in keeping with the Gompertzian model of increased cell proliferation in smaller tumours.

Currently p53 protein is thought to act as a 'genomic guardian' which arrests cells with DNA mutations at the $G_1$–S checkpoint and prevents cell replication with the propagation of the mutation (Lane, 1992; Vogelstein & Kinzler, 1992). Treatment of cells *in vitro* with irradiation, ultraviolet light and radiomimetic drugs has been shown to induce accumulation of p53 in a response to DNA damage (Wynford-Thomas, 1992; Fritsche *et al*., 1993; Hall *et al*., 1993). Similar effects have been found in the skin *in vivo* following UV exposure (Hall *et al*., 1993). Initially the detection of p53 protein by immunohistochemistry was thought to be possible only when abnormally stabilised mutant protein was present. More recent reports have emphasised that accumulation of even wild-type p53 can also allow its immunohistochemical detection but that the function of the protein may still be abnormal (Walker *et al*., 1991). No results have been published of the effect of cytotoxic drugs administered *in vivo* on p53 expression. We interpret the acquisition of p53 staining in 8 of 30 cases as being the result of accumulation of normal protein as a result of gentoxic damage caused by the cytotoxic drugs and not new mutations in the p53 gene. Thus, this is suggested to be further support for the role of p53 in DNA repair in a human tumour *in vivo*.

### References

AKANUMA, A. (1978). Parameter analysis of gompertzian function growth model in clinical tumours. *Eur. J. Cancer*. **14**, 681–688.

BARNES, D.B., MEYER, J.S., GONZALEZ, J.G., GULLICK, W.J. & MILLIS, R.R. (1991). Relationship between c-*erb*B-2 immunoreactivity and thymidine labelling index in breast carcinoma *in situ*. *Breast Cancer Res. Treat*., **18**, 11–17.

BARNES, D.B., DUBLIN, E.A., FISHER, C.F., LEVISON, D.A. & MILLIS, R.R. (1993). Immunohistochemical detection of p53 protein in mammary carcinoma: an important new independent indicator of prognosis? *Hum. Pathol*., **24**, 469–476.

BONADONNA, G., VERONESI, V., BRAMBILLA, C., FERRARI, L., LUINI, A., GRECO, M., BARTOLI, C., COOPMANS DE YOLDA, G., ZUCALI, R., RILKE, F., ANDREOLA, S., SILVESTRINI, R., DI FRONZO, G. & VALAGUSSA, P. (1990). Primary chemotherapy to avoid mastectomy in tumours with diameters of three centimetres or more. *J. Natl Cancer Inst*., **82**, 1539–1545.

BRIFFORD, M., SPYRATOS, F., TUBIANA-HUHN, M., PALLUD, C., MAYRAS, C., FILLEUL, A. & ROUESSE, J. (1989). Sequential cytopunctures during pre-operative chemotherapy for primary breast cancer. *Cancer*, **63**, 631–637.

CAMPLEJOHN, R.S., BROCK, A., BARNES, D.B., GILLETT, C., RAIKUNDALIA, B., KREIPE, H. & PARWARWSCH, M.R. (1993). Ki-S1, a novel proliferative marker: flow cytometric assessment of staining in human breast carcinoma cells. *Br. J. Cancer*, **67**, 657–662.

CATTORETTI, G., BECKER, M.H.G., KEY, G., DUCHROW, M., SCHLUTER, C., GALLE, J. & GERDES, J. (1992). Monoclonal antibodies against recombinant parts of the Ki-67 antigen (MIB1 and MIB3) detect proliferating cells in microwave processed, formalin-fixed, paraffin sections. *J. Pathol*., **168**, 357–363.

CHAN, W.K., POULSOM, R., LONG, Q.-L., PATEL, K., GREGORY, W., FISHER, C.F., LEVISON, D.A. & HANBY, A.M. (1993*a*). *Bcl*-2 expression in invasive mammary carcinoma: correlation with apoptosis, hormone receptors and p53 expression. *J. Pathol*. (Suppl.), **169**, 153A.

CHAN, W.K., POULSOM, R., LONG, Q.-L., GREGORY, W., FISHER, C.F., LEVISON, D.A. & HANBY, A.M. (1993*b*). *Bcl*-2 expression in invasive mammary carcinoma: correlation with apoptosis, tumour grade and hormone receptor status. *J. Pathol*. (in press).

DIVE, C. & WYLLIE, A.H. (1992). Apoptosis and cancer chemotherapy. In *Frontiers in Pharmacology: Cancer Chemotherapy*, Hickman, J.A. & Tritton, T.T. (eds). Blackwell Scientific Publications: Oxford.

DYSON, J.E.D., SIMMONS, D.M., DANIEL, J., MCLAUGHLIN, J.M., QUIRKE, P. & BIRD, C.C. (1986). Kinetic and physical studies of cell death induced by chemotherapeutic agents or hyperthermia. *Cell Tissue Kinet*., **19**, 311–324.

EARLY BREAST CANCER TRIALISTS' COLLABORATIVE GROUP (1992). Systemic treatment of early breast cancer by hormonal, cytotoxic or immune therapy. *Lancet*, **339**, 1–15, 71–85.

EASTMAN, A. (1990). Activation of programmed cell death by anticancer agents: cisplatin as a model system. *Cancer Cells*, **2**, 275–280.

FORNI, A.M., KOSS, L.G. & GELLER, W. (1964). Cytologic study of the effects of cyclophosphamide on the epithelium of the urinary bladder in Man. *Cancer*, **17**, 1348–1355.

FRITSCHE, M., HAESSLER, C. & BRANDNER, G. (1993). Induction of nuclear accumulation of the tumour-suppressor protein p53 by DNA-damaging agents. *Oncogene*, **8**, 307–318.

HALL, P.A., MCKEE, P.H.M., MENAGE, H., DU, P., DOVER, R. & LANE, D.P. (1993). High levels of p53 protein in UV irradiated normal human skin. *Oncogene*, **8**, 203–207.

KASTAN, M.B., ONYEKWERE, O., SIDRANSKY, D., VOGELSTEIN, B. & CRAIG, R.W. (1991). Participation of p53 protein in the cellular response to DNA damage. *Cancer Res*., **51**, 6304–6311.

KENNEDY, S.M., MERINO, M.J., SWAIN, S., LIPPMAN, M.E. & TAYLOR, S.R. (1989). Breast cancer kinetics and ploidy before and after chemotherapy. *Lab. Invest*., **60**, 47A.

KENNEDY, S., MERINO, M.J., SWAIN, S., LIPPMAN, M.E. (1990). The effects of hormonal and chemotherapy on tumoural and non-neoplastic breast tissue. *Hum. Pathol*., **21**, 192–198.

KYPRIANOU, N., ENGLISH, H.F., DAVIDSON, N.E. & ISAACS, J.T. (1991). Programmed cell death during regression of the MCF-7 human breast cancer following oestrogen ablation. *Cancer Res.*, **51**, 162–166.

LAIRD, A.K. (1964). Dynamics of tumour growth. *Br. J. Cancer*, **18**, 490–502.

LANE, D.P. (1992). p53, guardian of the genome. *Nature*, **358**, 15–16.

LENNON, S.V., MARTIN, S.J. & COTTER, T.G. (1990). Induction of apoptosis (programmed cell death) in tumour cell lines by widely diverging stimuli. *Biochem. Soc. Trans.*, **18**, 343–346.

McCORMICK, D., YU, C., HOBBS, C. & HALL, P.A. (1993). The relevance of antibody concentration to the immunohistological quantification of cell proliferation-associated antigens. *Histopathology*, **22**, 543–547.

NATHAN, B., ANBAZHAGAN, R., DYER, M., EBBS, S.R., JAYATILAKE, H. & GUSTERSON, B.A. (1993). Expression of *bcl*-2 like immunoreactivity in breast cancer. *Breast*, **2**, 134–137.

O'REILLY, S., CAMPLEJOHN, R.S., RUBENS, R.D. & RICHARDS, M.A. (1992). DNA flow cytometry and response to pre-operative chemotherapy for primary breast cancer. *Eur. J. Cancer*, **28**, 681–683.

PAIK, S., HAZAN, R., FISHER, E.R, SASS, R.E., FISHER, B., REDMOND, C., SCHLESSINGER, J., LIPPMAN, M.E. & KING, C.R. (1990). Pathologic findings from the National Surgical Adjuvant Breast and Bowel Project: Prognostic significance of c-*erb*B-2 protein overexpression in primary breast cancer. *J. Clin. Oncol.*, **8**, 103–112.

SAMPSON, S.A., KREIPE, H., GILLETT, C., SMITH, P., CHAUDARY, M.A., KHAN, A., PRAWARESCH, R. & BARNES, D.B. (1992). Ki-S1 – a novel marker of cell proliferation and its relationship to prognosis in mammary carcinoma. *J. Pathol.*, **168**, 179–185.

SEARLE, J., LAWSON, P.J., ABBOTT, P.J., HARMON, B. & KERR, J.F.R. (1975). An electron-microscope study of the mode of cell death induced by cancer-chemotherapeutic agents in populations of proliferating normal and neoplastic cells. *J. Pathol.*, **116**, 129–138.

SKIPPER, H.E., SCHABEL, F.M. & WILCOX, W.S. (1964). Experimental evaluation of potential anti-cancer agents. XIII. On the criteria and kinetics associated with curability of experimental leukaemia. *Cancer Chemother. Rep.*, **35**, 1–111.

SKOOG, L., RUTQVIST, L.E. & WILKING, N. (1992). Analysis of hormone receptors and proliferation fraction in fine needle aspirates from primary breast carcinomas during chemotherapy or tamoxifen treatment. *Acta Oncol.*, **31**, 139–141.

VOGELSTEIN, B. & KINZLER, K.W. (1992). p53 function and dysfunction. *Cell*, **70**, 523–526.

WALKER, R.A., DEARING, S.J., LANE, D.P. & VARLEY, J.M. (1991). Expression of p53 protein in infiltrating and *in situ* breast carcinomas. *J. Pathol.*, **165**, 203–211.

WALLER, U. (1960). Giant nuclei after Myleran therapy and splenic irradiation. *Pathol. Microbiol.*, **23**, 283–290.

WHELAN, R.D.H., McCLEAN, S. & HILL, B.T. (1992). Drug resistant MCF-7 cells selected following exposure to fractionated X-irradiation retain functional steroid and epidermal growth factor receptors and express pS2 but not c-*erb*B-2 (abstract). *Proc. Am. Assoc. Cancer Res.*, **33**, 454.

WYLLIE, A.H. (1993). Apoptosis. *Br. J. Cancer*, **67**, 205–208.

WYNFORD-THOMAS, D. (1992). p53 in tumour pathology: can we trust immuno-cytochemistry? *J. Pathol.*, **166**, 329–330.